# Characterization of the complete chloroplast genome of *Saussurea involuerata* (Compositae), an endangered species endemic to China

**DOI:** 10.1080/23802359.2019.1705195

**Published:** 2020-01-10

**Authors:** Rui Wang, Jinfeng Liu, Siyan Liu, Shuyan Guan, Peng Jiao

**Affiliations:** College of Life Sciences, Jilin Agricultural University, Changchun, Jilin, China

**Keywords:** *Saussurea involuerata*, endangered species, chloroplast genome

## Abstract

*Saussurea involuerata* (S. involuerata), a kind of composite and perennial herbage plant, is a rare and precious alpine medicinal plants with high medicinal value, it can regulate human’s body and has health care function, therefore attracts attention of many scholars. It is currently on the International Union for Conservation of Nature (IUCN) red list of threatened species. In this study, we first assembled the complete chloroplast (cp) genome of *S. involucrata* by Illumina paired-end reads data. The whole genome was 152,490 bp, consisting of a pair of inverted repeats of 69,041 bp, large and small single-copy regions of 69,045bp and 18,638 bp in length, respectively. The cp genome contained 134 genes, including 90 protein-coding genes, 36 tRNA genes, and 8 rRNA genes. The overall GC content of the whole genome was 37.7%. A neighbor-joining phylogenetic analysis demonstrated a close relationship between *S. involuerata* and *Arctium lappa*.

*Saussurea involuerata* (*S. involucrata*) is an alpine ice field herbaceous plant that blooms and bears perennial in the genus Echinacea. It grows on the steep mountain walls at 2400–4100 m above sea level in Tianshan Mountains, Xinjiang. It is a rare, slow-growing and very precious Chinese herbal medicine. It has the functions of dispersing cold and dehumidifying, promoting blood circulation, anti-inflammatory and analgesic, and contracting the uterus. It is mainly used for various rheumatoid arthropathy, rheumatism, and irregular menstruation. Studies have shown that Tianshan Snow Lotus contains flavonoids, phenylpropanoids, polysaccharides, alkaloids and lactones, and has pharmacological effects such as antioxidant, anti-inflammatory, anti-fatigue and anti-ischemia/reperfusion injury (Zhai et al. 2010; Chik et al. 2015). In 1996, China has listed *S. involuerata* as a national second-level protected plant. Its natural reproduction rate is low, artificial cultivation is difficult, and long-term predatory mining makes its wild resources endangered.

In this study, *S. involuerata* was sampled from the Endangered Species Reserve of Jilin Agricultural University Changchun County, China (125°19′E, 43°43′N). A voucher specimen (WR20191204) was deposited in the Herbarium of the Plant Biotechnology Center of Jilin Agricultural University, Changchun, China.

The present study is the first time to assemble and characterize the complete chloroplast genome for *S. involuerata* (GenBank: NC_029465.1) from high-throughput sequencing data. The existing chloroplast Genome sequence of *S. involucrata* was downloaded from the National Center for Biotechnology Information’s Organelle Genome Resources database (NC_029465.1) as the reference sequence, and the chloroplast Genome of *S. involucrata* was assembled using SPAdes v3.6.0 software (Bankevich et al. 2012). The default setting of parameters was adopted. Sequence annotation first confirmed the availability and boundary of genes by BlastN comparison directly through the protein-coding sequence of the proximal species. Then, the genes in the chloroplast genome were annotated by online tool DOGMA (http://dogma.ccbb.utexas.edu/) with default parameters, and the genes were functionally annotated by combining with NR (http://www.ncbi.nlm.nih.gov/) database (Lohse et al. 2013). TRNA was annotated using the trnascanse online site. Using RNAmmer 1.2 Server (http//www.cbs.dtu.dk/services/RNAmmer/) rRNA for comments (Katoh and Standley 2013). The chloroplast genome of S. involucrata was mapped using OGDRAW (HTTP//OGDRAW. Mpimp-golm. mpg. DE/cgibin/OGDRAW. Pl) software (Asaf et al. 2017).

The complete cp-DNA of S. involuerata was a circular molecule 152,490 bp in length, comprising a large single copy (LSC) region of 69,045 bp and a small single copy(SSC) region of 18,638 bp, separated by two inverted repeat regions (IRs) of 69,041 bp (Figure 1). It contained 134 genes, including 90 protein-coding genes, 8 ribosomal RNA genes, and 36 tRNA genes. The phylogenetic tree reveals all the species of Asteraceae formed a monophyletic clade with high-resolution value and *Arctium lappa* is highly related with *S. involuerata* (Figure 1).

**Figure 1.**
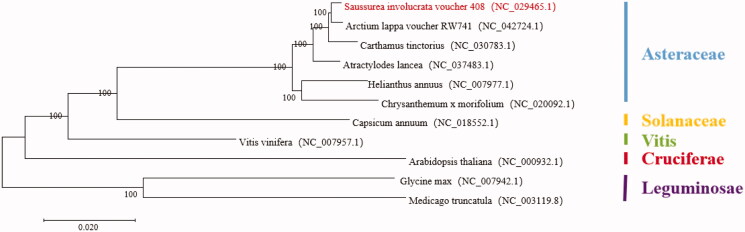
The phylogenetic tree based on 11 complete plastid genome sequences.
